# Control of pH-Responsiveness in Graphene Oxide Grafted with Poly-DEAEMA via Tailored Functionalization

**DOI:** 10.3390/nano10040614

**Published:** 2020-03-27

**Authors:** Roxana Noriega-Navarro, Jésica Castro-Medina, Martha V. Escárcega-Bobadilla, Gustavo A. Zelada-Guillén

**Affiliations:** 1 School of Chemistry, National Autonomous University of Mexico (UNAM), Circuito Escolar s/n, Ciudad Universitaria, Coyoacán, Mexico City 04510, Mexico; roxnn77@gmail.com (R.N.-N.); jesicm15@gmail.com (J.C.-M.); 2 Escuela Nacional Preparatoria 8 Miguel E. Schulz, Lomas de Plateros s/n, Álvaro Obregón, Mexico City 01600, Mexico

**Keywords:** graphene oxide, DEAEMA, radical polymerization, pH-sensitive materials, swelling ratio, potentiometry

## Abstract

Polymer-grafted nanomaterials based on carbon allotropes and their derivatives (graphene oxide (GO), etc.) are typically prepared by successive reaction stages that depend upon the initial functionalities in the nanostructure and the polymerization type needed for grafting. However, due to the multiple variables involved in the functionalization steps, it is commonly difficult to predict the properties in the final product and to correlate the material history with its final performance. In this work, we explored the steps needed to graft the carboxylic acid moieties in GO (COOH@GO) with a pH-sensitive polymer, poly[2-(diethylamino)ethyl methacrylate] (poly[DEAEMA]), varying the reactant ratios at each stage prior to polymerization. We studied the combinatorial relationship between these variables and the behavior of the novel grafted material GO-*g*-poly[DEAEMA], in terms of swelling ratio vs. pH (%Q) in solid specimens and potentiometric response vs. Log[H^+^] in a solid-state sensor format. We first introduced *N*-hydroxysuccinimide (NHS)-ester moieties at the –COOH groups (GO-*g*-NHS) by a classical activation with *N*-ethyl-*N*′-(3-dimethylaminopropyl)carbodiimide (EDC). Then, we substituted the NHS-ester groups by polymerizable amide-linked acrylic moieties using 2-aminoethyl methacrylate (AEMA) at different ratios to finally introduce the polymer chains via radical polymerization in an excess of DEAEMA monomer. We found correlated trends in swelling pH range, interval of maximum and minimum swelling values, response in potentiometry and potentiometric linear range vs. Log[H^+^] and could establish their relationship with the combinatorial stoichiometries in synthetic stages.

## 1. Introduction

Nanostructured carbon allotropes such as carbon nanotubes [1] and graphene [2] are among the most versatile nanomaterials due to their useful chemical, mechanical and electrical properties: e.g., size-dependent interfacial reactivity/solubility, high Young’s modulus, excellent electrical conductivity, high double-layer capacitance, etc. [1,2,3,4,5,6,7]. These properties are mainly originated by their low-dimensionality (1D, 2D) and high surface-to-volume ratio as well as their π-conjugated surface [8], which have propelled their incorporation into a wide range of functional systems where they usually play a central role. The usage of these nanostructures commonly takes advantage of one or more of the different properties they possess, for instance, as nanometric supports for heterogeneous catalysts [9,10,11], optical or electrochemical transducers in sensors and biosensors [12,13,14,15], mechanical reinforcement or electrical percolation modulation in polymer nanocomposites [16,17,18,19,20], nanoscale-enhanced radiosensitization and drug delivery in cancer therapy and tumor bioimaging [21,22,23], among others. However, in order to provide them with the additional features required for a particular purpose (catalytic activity, ion recognition, stimuli responsiveness, etc.), these nanostructures are usually converted into their chemically hybrid counterparts through successive synthetic modification steps (chemical grafting) that depend upon the departing nanomaterial, the functional molecule to be grafted to the latter and the combination of final properties required in the hybrid structure. 

In this sense, the design of carbon nanostructures covalently grafted with macromolecules such as synthetic polymers, oligonucleotides or proteins is a useful strategy to produce hybrid nanomaterials with a combination of features that are provided by both components [24,25]. As an example, graphene derivatives containing superficial reactive sites, such as –OH or –COOH in graphene oxide (GO), can be chemically grafted, on the one hand, with stimuli-responsive acrylic polymers to yield pH- and temperature-sensitive nanomaterials exhibiting external triggering in their set of properties, such as swelling, hydrophobicity and electrical conductivity [26,27]. On the other hand, grafting can be similarly carried out with ion recognition elements to generate hybrid ion-to-electron transduction/recognition components in potentiometric sensors [12,13]. Nevertheless, in all cases, it is necessary to first introduce superficial reactive sites at the starting carbonaceous material via suitable chemical reactions such as carboxylation, hydroxylation, amidation, esterification or silanization [25,28,29], commonly through intermediate activation steps (e.g., the classical use of carbodiimides prior to the formation of labile esters with *N*-hydroxysuccinimide for further amidation of carboxylated nanocarbons [30,31,32]). In this way, the range of useful reactive groups that can be introduced to the carbon nanomaterial surface, for example, for further polymer grafting, is as large as the polymerization chemistries available in the literature, which rely on three approaches [33]: (i) grafting through, that consists of polymer chains incorporated into the superficial reactive groups through the propagation reaction; (ii) grafting from, i.e., propagation of the polymer chains from surface-attached initiators; and (iii) grafting to, or attachment of, previously synthesized polymer chains to the reactive groups. For instance, vinylic or acrylic moieties can be superficially incorporated to the nanocarbons for further grafting through radical polymerization [16,34,35], alkyl halides or alkyl trithiocarbonates can be, respectively, used in grafting from for successive atom transfer radical polymerization (ATRP) [36] or reversible addition–fragmentation chain-transfer (RAFT) [37] polymerization and azido-terminated polymers can be otherwise exploited in grafting to using click chemistry [38,39].

Nowadays, there has been a steady increase in the use of GO instead of pristine graphene for the design of functional nanostructured hybrid systems. This trend has been maintained for the past few years because GO presents a higher solubility in water and simpler chemical derivatization in comparison with its pristine counterpart [40]. In the laboratory, the as-produced GO usually exhibits a wide range of reactive groups such as epoxy, anhydride, –OH or –COOH [41]. However, it is currently possible to achieve varieties with a major content of carboxylic groups and negligible amounts of the rest ones (namely, carboxylated GO) [42,43,44,45], thus opening the opportunity to finely tuned derivatization schemes that are crucial in more complex systems as in polymer/GO hybrids. 

In this regard, pH-sensitive polymer/GO hybrids are appealing complex systems that currently find direct applications as smart nano-objects or interactive materials in the fields of sensors, diagnostics and therapeutics [46]. In these systems, the polymer grafts are used as a triggering component that responds upon external stimuli, for instance, when pH values rise to a point at which the polymer can undergo a phase change such as charged/swollen-to-neutral/shrunk and vice versa. This property has been exploited in polymer/GO hybrids to achieve, for example, on/off aflatoxin amperometric sensors [47] and externally triggered the release of cargo molecules such as anticancer drugs by near infrared-light phototherapy or local pH variations in tumors [48,49,50]. Therefore, the design of systems derived from GO and pH-responsive polymers is an attractive scenario not only in the current fabrication of portfolios with a wide set of input/output properties (external stimuli/internal response), but also as experimental platforms in the future creation of materials by machine learning strategies in which, nowadays, the required big data sets of synthesis conditions/input/output properties are scarce [51,52].

However, as mentioned earlier, successive modification steps must be implemented with the aim of providing an optimal combination in the final properties in the nanomaterial, which means that a controlled performance through tailored functionalization is usually a complicated task. In this work, we designed a new hybrid nanomaterial GO-*g*-poly[DEAEMA] with tunable pH-sensitive swelling and potentiometric transducing properties, by varying the reactant ratios at each stage prior to polymerization. The latter was performed by exploiting the –COOH groups at carboxylated GO (COOH@GO) which were first activated with *N*-ethyl-*N*′-(3 dimethylaminopropyl)carbodiimide (EDC) and esterified with *N*-hydroxysuccinimide (NHS) to yield the GO-*g*-NHS intermediate material in a synthetic Stage I. The NHS-ester groups formed were then exploited to carry out a classical amidation via nucleophilic substitution with 2-aminoethyl methacrylate (AEMA) to generate the GO-*g*-AEMA material in a synthetic Stage II. Finally, the functional hybrid material polymer/GO was synthesized in a synthetic Stage III by grafting through at the linked AEMA moieties with the stimuli-responsive polymer poly[2-(diethylamino)ethyl methacrylate] (poly[DEAEMA]) via radical polymerization in an excess of DEAEMA monomer and azobisisobutyronitrile (AIBN) as initiator.

## 2. Materials and Methods

### 2.1. Materials, Synthesis and Characterization

#### 2.1.1. General

All reagents and solvents were used as received, unless otherwise stated. 2-(*N*-morpholino) ethanesulfonic acid (MES; 99%), *N*-(3-dimethylaminopropyl)-*N*′-ethylcarbodiimide hydrochloride (EDC; 98%), *N*-hydroxysuccinimide (NHS; 98%), Na_2_HPO_4_·12H_2_O (99%), NaH_2_PO_4_·2H_2_O (99%), 2-aminoethyl methacrylate hydrochloride (AEMA; 90%), azobisisobutyronitrile (AIBN; 12 wt % in acetone), 2-(diethylamino)ethyl methacrylate (DEAEMA; 99 %), tetrahydrofuran (THF; 99%) and carboxylated graphene oxide (GO; 4.09 mmol COOH/g and 75.3%C content according to provider specifications) used in synthesis Stages I, II and III were provided by Sigma-Aldrich Co. (Saint-Louis, MI, USA). N_2_ (99.9 9%) was purchased from Infra (Mexico City, Mexico). Prior to use, DEAEMA was purified using a silica gel preparative column and then stored at 4 °C until needed. Deionized water obtained from a Thermo Scientific Barnstead Nanopure deionization apparatus (Thermo Fisher Scientific Inc., Waltham, MA, USA) was used to prepare all the buffer solutions employed in the synthesis and in the performance evaluations. 

#### 2.1.2. Buffer Solutions

pH buffer solutions for performance evaluations were prepared using KCl as background for ionic strength adjustment (50 mM), departing from stock solutions of a suitable buffering salt (1 M) and diluted with additional background KCl solution to a final concentration of 5 mM in the respective salt: sodium dihydrogen citrate (NaH_2_Cit; 99%, for pH = 3.0), potassium acetate (KOAc; 99%, pH = 4.0 and 5.0), MES (99%, pH = 6.0), NaH_2_PO_4_·2H_2_O, Na_2_HPO_4_·12H_2_O and/or Na_3_PO_4_·12H_2_O at different ratios (99%, pH = 7.0 to 11.0). All the buffer solutions were first purged with N_2_ for 5 min and then pH-adjusted using a model pH 2700 Benchtop Meter with pH electrode (Oakton Instruments, Vernon Hills, IL, USA) by addition of drops of either 1 M HCl or 1 M NaOH as needed. 

#### 2.1.3. Dispersion of GO Materials

Dispersions of aqueous GO solutions (or their derivatives) were accordingly done using a Sonopuls mini20 ultrasonic homogenizer coupled with a 2.5 mm diameter tip (Bandelin electronic GmbH & Co. KG, Berlin, Germany), for 15 min, 70% input and 0.5 Hz, at room temperature, whereas mixing of solutions was done with a standard vortex mixer. Dispersions of GO-*g*-AEMA in the DEAEMA monomer were performed in a model Sonorex Digitec ultrasonic bath (Bandelin electronic GmbH & Co. KG, Berlin, Germany), for 15 min. Thermal control of reactions was done with a model Pura 4 water bath (Julabo GmbH, Seelbach, Germany) coupled to a test tube rack. 

#### 2.1.4. Analysis of GO Materials

Thermogravimetric analyses (TGA) were done under synthetic air flow with a ramp of 10 °C/min on an SDT-Q600 TA Instruments apparatus (TA Instruments, Inc., New Castle, DE, USA); starting temperature 30 °C, final temperature 1000 °C. Fourier-transform infrared (FT–IR) spectra of products were recorded on an FTIR Spectrum RXI spectrophotometer (Perkin Elmer Inc., Waltham, MA, USA) using the attenuated total reflectance (ATR) technique; the spectra were scanned at a resolution of 1.0 cm^–1^. Scanning electron microscopy (SEM) was carried out at 10^–6^ Torr and 20 kV using a JSM-5900 microscope (Jeol Ltd. Tokyo, Japan) and an Oxford AZtec EDS detection system; solid samples were analyzed at the microscope without any previous treatment. High-resolution transmission electron microscopy (HR–TEM) analysis was performed on a JEOL model JEM-2010 microscope (Jeol Ltd. Tokyo, Japan) with an acceleration voltage of 200 keV, 50-micrometer C2 aperture, spot size 1 and variable dose rate. Images were processed with Gatan Digital Micrograph software (Gatan, Inc., Pleasanton, CA, USA) and analyzed with ImageJ (National Institutes of Health, Bethesda, MD, USA); samples were prepared by drop-casting 5 mg/mL solutions of GO derivatives in ethanol on formvar carbon film-covered square mesh copper grids and dried completely for 4 h at 298 K. 

#### 2.1.5. Potentiometric Measurements

Potentiometric measurements were carried out using a precision electrochemistry electromotive force (EMF) interface with 8-independent high-impedance channels at 10^13^ Ω, model EMF-16 (Lawson Labs, Inc., Malvern, PA, USA), whereas the electromotive force data were recorded using the software Data Acquisition and Instrument Controller, DAIC, version L-EMF DAQ 3.0, provided by Lawson Labs, Inc. as well. Potentiometric determinations were carried out under a 2-electrode format, using an Ag/AgCl/KCl (3 M) double junction reference electrode with 1 M KCl electrolyte bridge model InLab Reference (Mettler Toledo, LLC, Columbus, OH, USA).

#### 2.1.6. Synthesis Stage I

Preparation of GO-*g*-NHS (NHS-ester derivative) was performed according to the literature with some adaptations as mentioned ahead [13,31,32,53]. In a typical experiment, 80 mg of GO was dispersed in a 3 mL MES buffer (50 mM, pH = 5) in an assay tube. The dispersion was then mixed with 1 mL of additional MES buffer containing the EDC necessary to have nominal molar ratios in EDC vs. COOH groups (EDC:COOH@GO) of 6 mol/mol (383 mg, 1.96 mmol), 4 mol/mol (256 mg, 1.31 mmol) or 2 mol/mol (127 mg, 0.65 mmol). The mixtures were then protected with parafilm and incubated without stirring at 25 °C for 20 min. Afterward, 0.5 mL of MES buffer including NHS at 2:1 molar ratio vs. EDC (NHS:EDC) was thoroughly mixed with the reaction and incubated for 40 min at 25 °C under stirring; NHS additions: 461 mg (3.93 mmol), 308 mg (2.62 mmol) or 154 mg (1.31 mmol), accordingly. The product was extensively washed with deionized water using a Millipore vacuum filtration system and a 47 mm diameter polyethersulfone (PES) membrane filter with a 0.45 μm pore size (Gelman Sciences Inc., Ann Arbor, Michigan, USA). The black solid was recovered and dried overnight at 80 °C until constant weight. Larger quantities were prepared by serial batch production following the last procedure. 

T_d_ (TGA): T_p1_ = 212 °C (residual carboxylic groups in GO), T_p2_ = 322 °C (grafted NHS-ester), T_p3_ = 685 °C (C-basis in GO), T_o_ = 182 °C, T_f_ = 707 °C. FT–IR (ATR)/cm^–1^: 3781 and 3335 (–OH), 2918 and 2849 (–CH_2_–), 1693 (C=O), 1574 (C=C), 1539 (C–N), 1057 (C–O), shown in Supplementary Figures S1 and S2.

#### 2.1.7. Synthesis Stage II

Preparation of GO-*g*-AEMA (AEMA-amide derivative) was carried out as described elsewhere with the following adaptations [53,54,55]. Typically, 70 mg of freshly prepared and dry GO-*g*-NHS were dispersed in 3 mL of a 50 mM pH = 7.2 phosphate buffer solution (PBS) in an assay tube. The dispersions were combinatorially mixed with variable amounts of AEMA in 3 mL PBS to reach AEMA:GO-*g*-NHS ratios equivalent to 6 mmol/g (77 mg, 0.42 mmol), 12 mmol/g (155 mg, 0.84 mmol) or 24 mmol/g (309 mg, 1.68 mmol), for each initial ratio EDC:COOH@GO obtained in Stage I, to yield nine possible combinations thereof. The reactions were incubated overnight at 25 °C under stirring conditions and protected from direct light. The product was filtered using a Nylon membrane (47 mm diameter and 0.45 μm pore size, GVS North America, Sanford, ME, USA), rinsed extensively with deionized water, dried overnight at 80 °C (dark gray solid) and stored at room temperature away from light. Larger amounts were prepared by serial batch production as well. 

T_d_ (TGA): T_p1_ = 206 °C (residual carboxylic groups in GO), T_p2_ = 335 °C (grafted AEMA-amide), T_p3_ = 771 °C (C-basis in GO), T_o_ = 164 °C, T_f_ = 779 °C. FT–IR (ATR)/cm^–1^: 3732 and 3419 (–OH), 2922 (–C–H aliphatic), 1681 and 1665 (C=O), 1574 (C=C), 1558 (C–N amide), 1139 (C–O), shown in Supplementary Figure S3.

#### 2.1.8. Synthesis Stage III

*Grafting through* of GO-*g*-AEMA to yield GO-*g*-poly[DEAEMA] was done via radical polymerization following a protocol adapted from the literature as mentioned ahead [16,29]. In a standard experiment, 60 mg of freshly synthesized and dry GO-*g*-AEMA were dispersed in a 5-fold weight excess of DEAEMA monomer (325 μL, 300 mg) and purged with N_2_ for 2 min. Immediately, 54 μL of AIBN solution (1.72 wt % vs. DEAEMA) was mixed with the dispersion and incubated for 5 h at 75 °C in an N_2_ atmosphere and without stirring. The solid product (black-bright and rubbery) was extensively washed with THF in a Nylon membrane filter to get rid of unbound polymer chains and unreacted monomer, dried overnight at 80 °C until constant weight and stored under vacuum in a standard laboratory desiccator until needed. Nine families of hybrid materials (polymer/GO) were accordingly obtained in this manner. Larger amounts were obtained by serial batch production. 

T_d_ (TGA): T_p1_ = 235 °C (labile oxygenated groups in the hybrid material polymer/GO), T_p2_ = 329 °C (residual aliphatic polymer content), T_p3_ = 646 °C (C-basis in GO component), T_o_ = 194 °C, T_f_ = 676 °C. FT–IR (ATR)/cm^–1^: 3416 (-OH), 2869 (C–H aliphatic), 1703 and 1653 (C=O), 1578 (C=C), 1556 (C–N amide), 1262 (C–N amine), 1140 (C–O), shown in Supplementary Figure S4.

### 2.2. Performance Evaluations

#### 2.2.1. Swelling Studies

In a typical assessment of material swelling vs. pH, a sample (ca. 10–20 mg) from one of the nine families of GO-*g*-poly[DEAEMA] was individually soaked into 1 mL of the pH buffer in the evaluation and incubated at 25 °C. After 1 h of incubation, equilibrium was reached and the solid was precipitated by centrifugation (13,000 rpm, 3 min), the supernatant was totally discarded and the excess of liquid over the solid pellet was carefully eliminated with a filter paper. The percentage of swelling ratio (%Q) was then evaluated by gravimetry. From the data, we determined the pH range at which the maximum (%Q_max_) and minimum (%Q_min_) swelling ratios occur for that material family (swelling pH range). Similarly, we also determined Δ[%Q] as the difference in swelling between %Q_max_ and %Q_min_ (the interval of maximum and minimum swelling values). The process was done by triplicate for each family at pH values between 3.0 and 11.0, whereas values beyond that range were not tested, according to preliminary evaluations that suggested hydrolysis in the materials (dramatic loss in weight). Blank evaluations were carried out as described above, using GO-*g*-AEMA instead of the polymer-grafted counterparts; blank prepared in Stage I with EDC:COOH@GO = 4 mol/mol and in Stage II with AEMA:GO-*g*-NHS = 24 mmol/g.

#### 2.2.2. Potentiometric Studies

Potentiometric response vs. pH was evaluated in the materials using the zero-current potentiometry technique by recording the electromotive force (EMF) between a working electrode prepared with the material family under assessment and the reference electrode described previously. In general terms, ca. 50 mg of one of the nine families of GO-*g*-poly[DEAEMA] were dispersed in 10 mL THF and deposited on a Nylon membrane filter (0.45 μm pore size) using extensive vacuum and further mechanical pressure when needed. The membrane was dried at room temperature for 24 h in a desiccator. After this procedure, a section containing the deposited material (ca. 50 μm thickness) was cut in stripes (0.5 cm × 2 cm) and these were individually used as solid-state sensors in further evaluations. Afterwards, freshly prepared sensors were directly connected to the high-impedance EMF interface by one of the ends and evaluated as described ahead. 

All the EMF measurements were carried out in individual cells containing 50 mL of the pH buffer in evaluation at 25 °C, by controlling the area of the respective electrode being exposed to the buffer to 0.5 cm^2^, and following the variations in the recorded values. The changes in EMF produced by stepwise shifts in pH from 11.0 to 3.0 (i.e., [H^+^] = 10^–11^ mol/L to [H^+^] = 10^–3^ mol/L) were monitored using the DAIC software until a constant plateau was observed in EMF vs. time (τ), e.g., when the slope δEMF/δτ ≈ 0.0 mV/h (stabilized EMF at pH 11.0 was used as initial baseline). The later stabilization signal was reached after 11.4 min in average for the specimens analyzed (±0.88 min at a 95% confidence interval for *N* = 240), while the change between the starting EMF value and the final value reached such plateau was considered as the 100% in response for each pH value (achieved at a response time τ_R100%(pH)_), therefore, for practical purposes, all the data were collected during 30 min for each pH. 

From the data, the potentiometric linear range in EMF vs. Log[H^+^] was determined and the potentiometric response was evaluated as the slope δEMF/δ(Log[H+]) in the linear range for each material family. Each material family was evaluated by triplicate at the pH values between 11.0 and 3.0 (prepared as described earlier), since initial explorations showed erratic behavior in response if testing was done at a wider range. Blank evaluations were carried out similarly using GO-*g*-AEMA in the whole process; blank was prepared in Stage I using EDC:COOH@GO = 4 mol/mol and in Stage II using AEMA:GO-*g*-NHS = 24 mmol/g. 

## 3. Results and Discussion

### 3.1. Material Evolution upon Synthesis Stage

**Figure 1:**
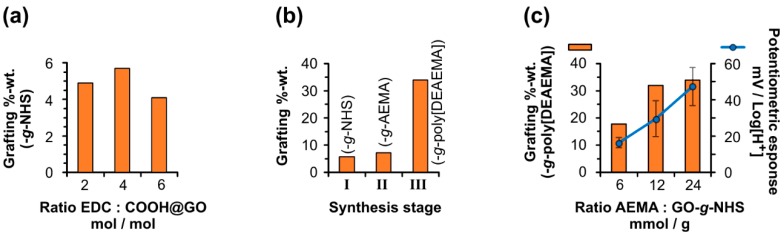
Grafting degree of graphene oxide (GO) depending on the reactant ratios at different stages: (**a**) *N*-hydroxysuccinimide (NHS)-ester amount introduced after varying *N*-ethyl-*N*′-(3-dimethylaminopropyl)carbodiimide (EDC) against the molar content of COOH per sample of GO (COOH@GO), for an NHS:EDC molar ratio = 2 in all the cases. (**b**) Material history in terms of graft content for a selected ratio EDC:COOH@GO of 4 mol/mol at Stage I, subsequently modified with amide-linked polymerizable 2-aminoethyl methacrylate (AEMA) moieties under a ratio of 24 mmol/g of AEMA vs. the departing material GO-*g*-NHS at Stage II, and its further polymerization with a five-fold excess in weight of 2-(diethylamino)ethyl methacrylate (DEAEMA) against GO-*g*-AEMA in Stage III. (**c**) Amount of poly[DEAEMA] chains incorporated to GO after Stage III for a fixed five-fold excess in weight of DEAEMA vs. GO-*g*-AEMA, but a variable ratio in AEMA vs. GO-*g*-NHS at Stage II and an initial molar ratio EDC:COOH@GO = 4 mol/mol at Stage I; in (**c**), a preliminary potentiometric evaluation of the material in a flexible solid-state sensor format showed a positive correlation between polymer grafting degree and electrochemical response against Log[H^+^].

An analysis of the functionalization degree after synthesis Stage I preliminarily showed that there was not an observable trend between the ratio of EDC vs. content of COOH in GO and the number of NHS-ester moieties introduced by the carbodiimide activation method (Figure 1a). In this regard, a slight maximum of 5.7 wt % of grafted NHS (confirmed by the emergence of the FT–IR bands 2918 cm^–1^ and 2849 cm^–1^ for –CH_2_–, 1693 cm^–1^ for C=O, 1539 cm^–1^ for C–N and 1057 cm^–1^ for C–O, cf. Supplementary Figures S1 vs. S2) was reached at 4 mol/mol EDC:COOH@GO. However, if such value is compared with the minimum grafting amount of 4.1 wt % achieved at 6 mol/mol and the intermediate value of 4.9 wt % at 2 mol/mol, it is reasonable to conclude, to this point, that there is not an important difference in functionalization degree between all these values. But, as will be shown ahead, such a minor difference in the amount of functionalization would represent an important modulator of performance in the materials once being grafted with poly[DEAEMA], thus exhibiting that the hybrid material behavior strongly depends on the material history. 

To explore this hypothesis, we first compared the evolution in graft content after each synthesis at Stages I, II and III, at the successive ratios of EDC:COOH@GO = 4 mol/mol for I, AEMA:GO-*g*-NHS = 24 mmol/g for II and a standard five-fold weight excess in DEAEMA monomer vs. GO-*g*-AEMA in III (Figure 1b). The analysis showed a slight increase in functionalization degree between Stage I and II, from 5.7 wt % to 7.1 wt %, which is coherent with the higher molecular weight of the grafted AEMA-amide (confirmed by the presence of FT–IR bands 2922 cm^–1^ for C–H, 1681 cm^–1^ and 1665 cm^–1^ for C=O, 1558 cm^–1^ for C–N and 1139 cm^–1^ for C–O, see Supplementary Figure S3), if compared to the NHS-ester moieties present in the material after Stage I. However, graft content dramatically increased up to 34 wt % after completing Stage III due to the introduction of polymer chains in the hybrid material GO-*g*-poly[DEAEMA], as confirmed by the emergence of the FT–IR bands at 2869 cm^–1^ for aliphatic C–H, 1703 cm^–1^ and 1653 cm^–1^ for C=O, and finally, 1262 cm^–1^ for C–N of amine (Supplementary Figure S4). 

In morphological terms, no important differences were observed by SEM and HR–TEM after each of these three stages (Supplementary Figures S5, S6, S7 and S8). Interestingly, the amount of grafted polymer (wt % vs. C-basis in GO) directly depended on the ratio AEMA:GO-*g*-NHS (mmol/g) used in Stage II for a same initial ratio EDC:COOH@GO of 4 mol/mol in Stage I (Figure 1c); in other words, the larger the ratio in AEMA vs. GO-*g*-NHS, the higher the grafting degree in the hybrid material GO-*g*-poly[DEAEMA], cf. 17.8 wt % of polymer content for 6 mmol/g AEMA:GO-*g*-NHS, 31.9 wt % for 12 mmol/g and 34 wt % for 24 mmol/g, respectively. Finally, a preliminary evaluation of these hybrid material families in terms of potentiometric response showed a direct dependence of the latter upon the polymer content in which the higher the amount of grafted poly[DEAEMA], the higher the response achieved in a material (cf. blue curve vs. orange bars in Figure 1c). Therefore, we decided to study the potentiometric performance of all the nine hybrid material families GO-*g*-poly[DEAEMA] that were combinatorically obtained by variations in reactant ratios (Stages I–II) and to compare them with their swelling behavior against pH.

### 3.2. Performance Evaluations of the Material Family

**Figure 2:**
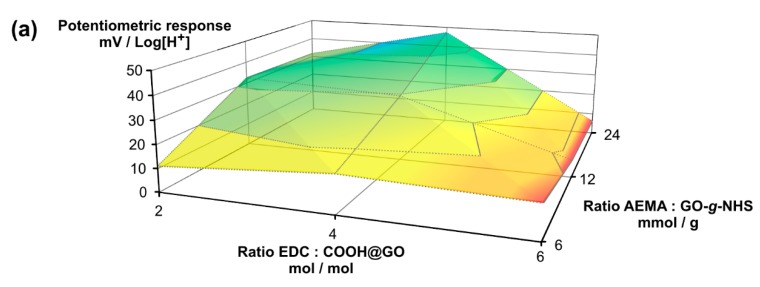

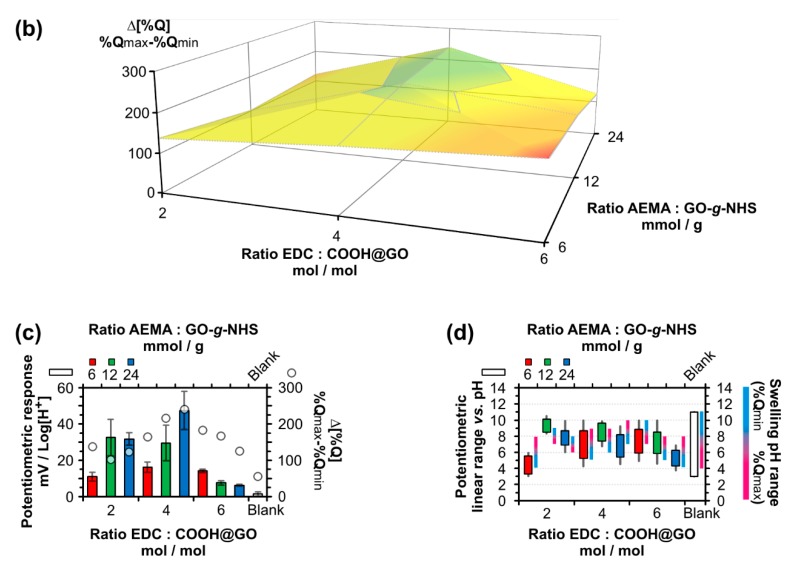
Performance evaluation for the nine different families of GO-*g*-poly[DEAEMA] obtained by combinatorial variations in reactant ratios. (**a**) Response surface curve for average potentiometric response against Log[H^+^]; (**b**) response surface curve for the difference between maximum and minimum average swelling ratios (Δ[%Q] = %Q_max_ – %Q_min_) for the same families in (**a**); (**c**) average potentiometric response values against Log[H^+^] including 1 s.d. in error bars, and their comparison with Δ[%Q]; (**d**) average potentiometric linear ranges for response vs. pH including 1 s.d. in error bars, and their comparison with the swelling pH ranges and routes at which minimum-to-maximum average swelling ratios occur. Average values and s.d. are for *N* = 3; blank materials in (**c**,**d**) are GO-*g*-AEMA.

From an analysis of the average response surface curves in both potentiometric response and Δ[%Q] in swelling studies, it is possible to observe a maximum in the former of 47.4 mV/Log[H^+^] (Figure 2a) and in the latter of 240% (Figure 2b), equally achieved for the same material family prepared at the combination of EDC:COOH@GO = 4 mol/mol and AEMA:GO-*g*-NHS = 24 mmol/g. In this sense, the simultaneous charting of these performance evaluations together with the data for our selected blank material (GO-*g*-AEMA, from 4 mol/mol EDC:COOH@GO vs. 24 mmol/g AEMA:GO-*g*-NHS) showed in Figure 2c a general degree of correlation between Δ[%Q] and potentiometric response in terms of the material family. There is a clearer trend, on the one hand, if the difference between the maximum and minimum average swelling ratio in the blank is compared with its potentiometric response (white bar and its respective white circle in Figure 2c) in the context of the rest of the points in the graph (red, green and blue bars and their corresponding circles in the same figure). In both cases, the lowest performance level is obtained in the blank, i.e., 1.5 mV/Log[H^+^] for potentiometric response and 56% for Δ[%Q], respectively. Similarly, it is possible to locally observe positive correlations between potentiometric response and Δ[%Q], in most of the datasets for the same EDC vs. carboxylic groups ratio, for example, at EDC:COOH@GO = 4 mol/mol or 6 mol/mol, where the higher the Δ[%Q] values, the larger the potentiometric response in each ratio. On the other hand, a similar assessment in the material families done in terms of potentiometric linear range in pH units and swelling pH range brings us to similar conclusions (Figure 2d). In that regard, potentiometric linear range values are relatively displaced to a similar degree as in their respective swelling pH ranges for each hybrid material family. For instance, in Figure 2d, the more acidic the lower (or the upper) pH limit of a linear range in potentiometry, the lower the initial (or final) pH at which swelling occurs. This is especially clearer for the series of EDC:COOH@GO = 2 mol/mol and 6 mol/mol. Similarly, the wider the linear range in potentiometry, the wider the swelling pH range for the latter. The latter is also easier to observe by comparing both trends in blank, where the potentiometric linear range comes from pH 11.0 to 3.0 and the swelling pH range happens from pH 11.0 to 4.0. Similar behavior is observed in the datasets for EDC:COOH@GO = 2 mol/mol and 4 mol/mol, where the red bars are larger than the green and blue ones and the same occurs with their respective swelling pH range bars. Although there is not an exact match between both parameters in all the datasets, the general trend strongly suggests two possible phenomena: (1) an interconnection between Δ[%Q] vs. pH and the change in EMF elicited by a stepwise increase in the concentration of [H^+^]; and (2) interdependence between the pH intervals at which the last two events occur.

### 3.3. Proposed Mechanism for pH-Dependence in the Material Performance

Nevertheless, there is no apparent connection between the potentiometric linear ranges and the swelling routes in each material (from the cyan segment in %Q_min_ to the magenta segment in %Q_max_ at swelling pH range bars in Figure 2d). However, this lack of correlation could be probably originated by the untypical swelling profile in the hybrid materials. Regarding this, all the swelling curves plotted vs. pH do not follow (in general) a classical sigmoidal shape as expected for a pH-sensitive polymer, but eventually exhibit local maximums in %Q of a lesser swelling degree than the highest value (%Q_max_) achieved for a material family, and thus, a non-linear behavior is mostly observed (Supplementary Figure S9). Such behavior could be explained if the hybrid material GO-*g*-poly[DEAEMA] is seen as a potentially zwitterionic system, where protonation and deprotonation processes occur both on the tertiary amine moieties at the polymer chains and on the remaining carboxylic groups at the surface of the GO portion, depending on the range of pH at which a material is being tested. In other words, according to the acid–base theory, it is expected that at the lowest pH values, both the carboxylic groups in GO and the tertiary amines in the polymer remain protonated. Using p*K*_a_ values reported in the literature as a reference guide, where p*K*_a_ for poly[DEAEMA] is described at 7.3 [56] and p*K*_a_ for COOH in GO is located at 4.11 and 6.50 [57], it is expected that only the alkyl ammonium moieties in the poly[DEAEMA] grafts are then responsible for swelling below pH ~4. On the other side, at the highest pH regimes, carboxylic moieties in GO exist under a deprotonated (and negatively charged) form, and they can also coexist with non-protonated polymer chains at ca. two pH units above the highest p*K*_a_ in the system, so the first ones should indeed promote swelling in the material above pH ~9. Depending on which of the last two scenarios contribute with a higher net charge in the material for the whole pH range tested, a maximum swelling capability then appears at a pH value either at the acidic or at the basic region (%Q_max_). Consequently, at intermediate pH regimes, it is reasonable to expect the simultaneous occurrence of negatively charged carboxylates in GO and positively charged poly[DEAEMA] chains (e.g., above pH ~4 and below pH ~9), which in sum and at a certain pH value, contribute with a global net charge that could potentially reach a minimum (isoelectric point) in the hybrid GO-*g*-poly[DEAEMA], and hence, a minimum in swelling is also reached (%Q_min_). In general, pH values for %Q_min_ in most of the hybrid materials fit in the last scenario, whereas the behavior in the blank also gives additional support to the proposed mechanism. Finally, the currently ongoing electrochemical studies are being carried out to unravel the ionic charge migration phenomena that would quantitatively explain at a physicochemical level all these trends, but so far, they fall out of the scope of this work.

## 4. Conclusions

To this point, it is clear that the presence of the poly[DEAEMA] chains is critical to provide the functionality to the hybrid system, but also that the material preparation history exerts an important role, since variations in reactant ratios result in strong differences in the overall final performances. However, this study opens the door for controlled responsiveness in our novel hybrid material GO-*g*-poly[DEAEMA] by simply selecting the suitable combination of ratios at Stages I and II, which, in turn, results in a simplified material design scenario using standard and well-known chemistry. Of course, orthogonal studies are needed to quantitatively determine the number of charges vs. pH in these materials so as to understand the whole complex phenomena involved herein, but, as a proof of concept, our findings demonstrate that a tailored functionalization is viable and that pH-responsiveness can be modulated in this manner. Finally, we believe that our findings could be useful in the future design of novel sensing platforms or drug delivery systems with tuned properties.

## Supplementary Materials

The following are available online at https://www.mdpi.com/2079-4991/10/4/614/s1, Figure S1: FT–IR spectrum of pristine GO, Figure S2: FT–IR spectrum of GO-*g*-NHS, Figure S3: FT–IR spectrum of GO-*g*-AEMA, Figure S4: FT–IR spectrum of GO-*g*-poly[DEAEMA], Figure S5: SEM of pristine GO and after synthetic Stage I, Figure S6: SEM of GO after synthetic Stage II and after Stage III, Figure S7: HR–TEM micrograph of pristine GO, Figure S8: HR–TEM micrograph of GO-*g*-poly[DEAEMA], Figure S9: Swelling curves against pH for GO-*g*-poly[DEAEMA] and blank.
