# Supplementary material for: Control of pH-Responsiveness in Graphene Oxide Grafted with Poly-DEAEMA via Tailored Functionalization
Source: Nanomaterials (Basel). 2020 Mar 27;10(4):614. doi: 10.3390/nano10040614 (PMC7221901; doi:10.3390/nano10040614)
Supplement: Supplementary file 1 [file nanomaterials-10-00614-s001.pdf]

Supplementary Information for:

Control of pH-responsiveness in graphene oxide grafted with poly-DEAEMA via tailored functionalization

Roxana Noriega-Navarro [1], Jésica Castro-Medina [1,2], Martha V. Escárcega-Bobadilla [1,*] and Gustavo A. Zelada-Guillén [1,*]

[1] School of Chemistry, National Autonomous University of Mexico (UNAM), Circuito Escolar s/n, Ciudad Universitaria, Coyoacán, Mexico City 04510, Mexico
[2] Escuela Nacional Preparatoria 8 Miguel E. Schulz, Lomas de Plateros s/n, Álvaro Obregón, Mexico City 01600, Mexico

***Correspondence**: mesbo@unam.mx (M.V.E.-B.); g.zelada@unam.mx (G.A.Z.-G.)

Table of contents:

Page S2: **Figure S1**. FT-IR spectrum of GO.
Page S2: **Figure S2**. FT-IR spectrum of GO-*g*-NHS.
Page S3: **Figure S3**. FT-IR spectrum of GO-*g*-AEMA.
Page S3: **Figure S4**. FT-IR spectrum of GO-*g*-poly[DEAEMA]
Page S4: **Figure S5**. SEM of pristine GO and after synthetic stage I.
Page S5: **Figure S6**. SEM of GO after synthetic stage II and after stage III.
Page S6: **Figure S7**. TEM micrograph of GO.
Page S6: **Figure S8**. TEM micrograph of GO-*g*-poly[DEAEMA].
Page S7: **Figure S9**. Swelling curves against pH for GO-*g*-poly[DEAEMA] and blank.

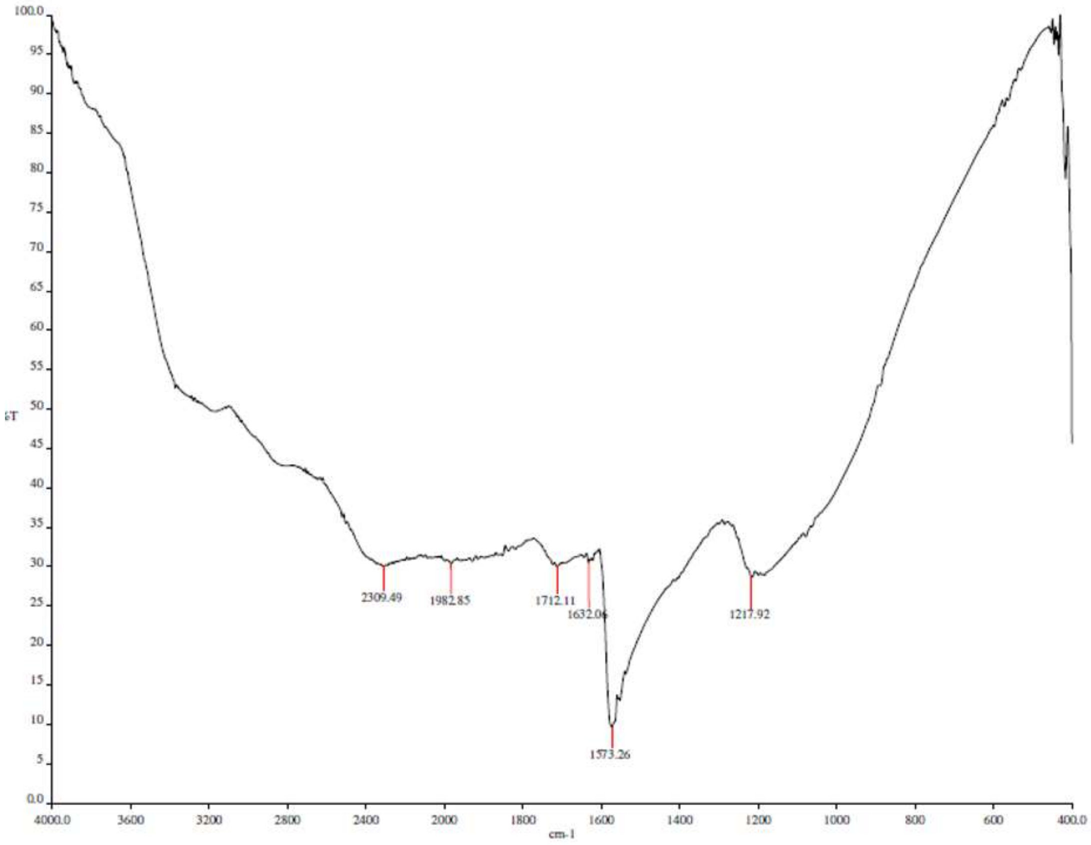

Figure S1. FT-IR spectrum of GO.

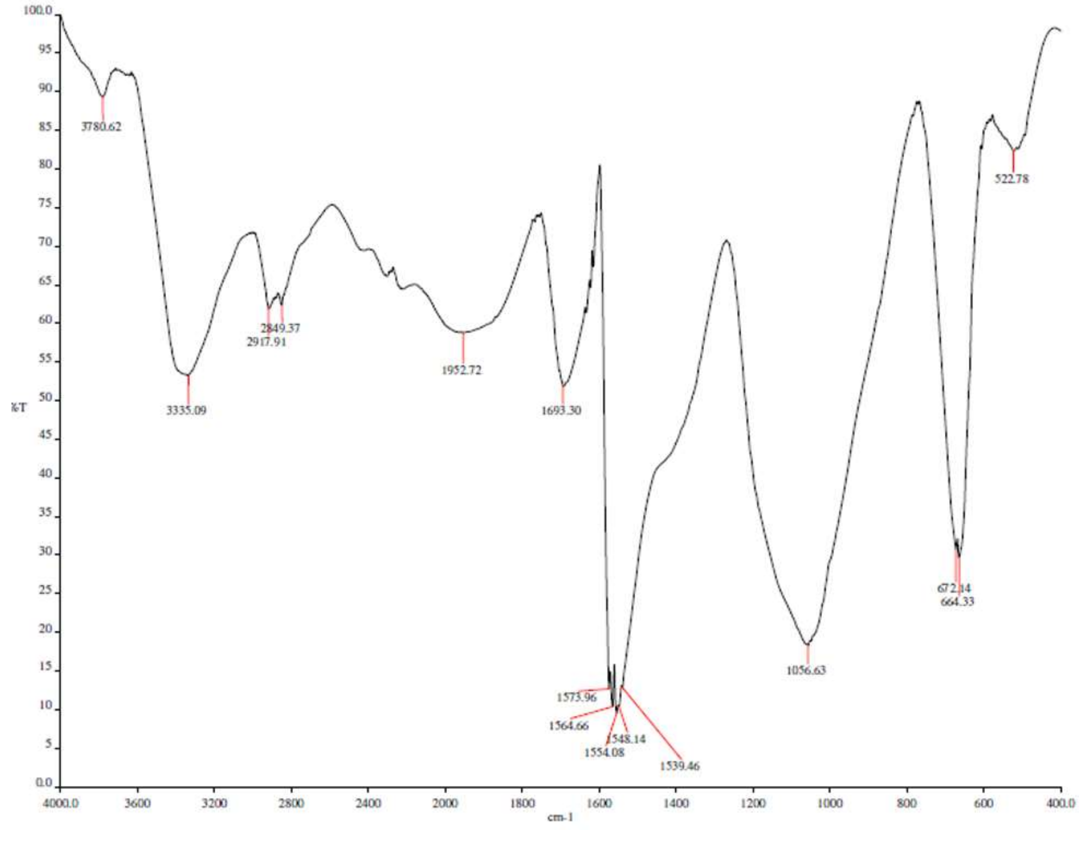

Figure S2. FT-IR spectrum of GO-*g*-NHS.

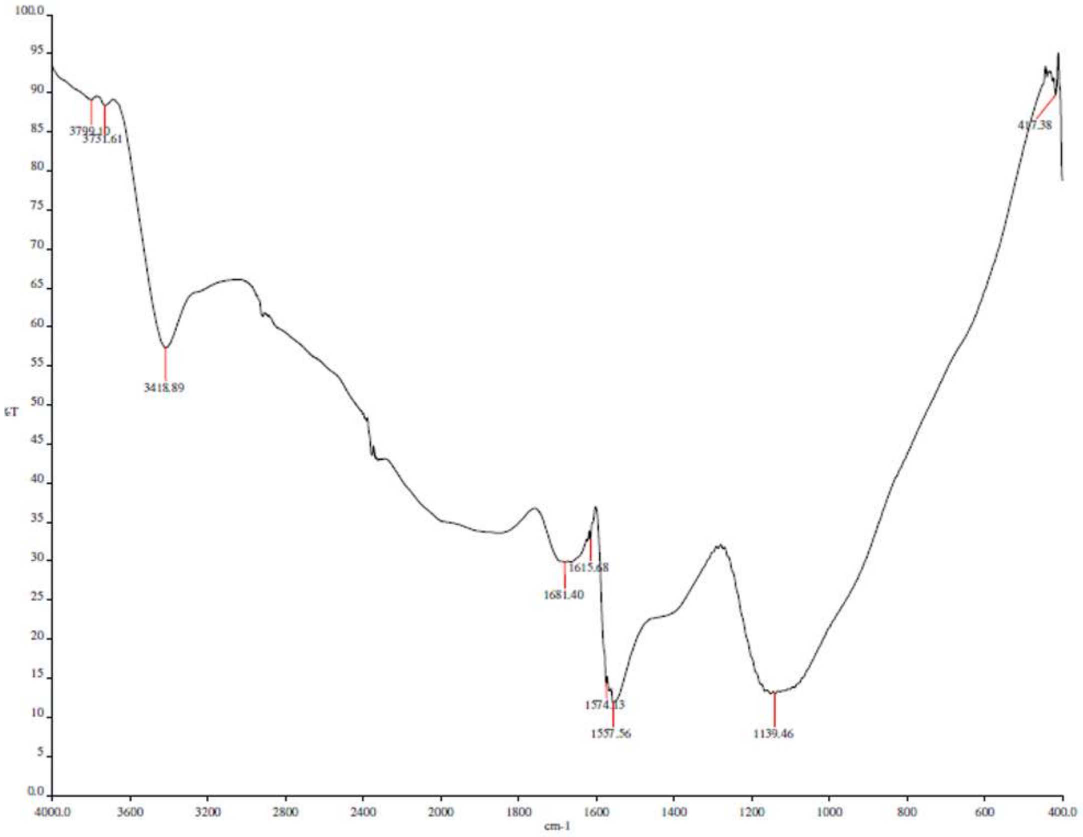

Figure S3. FT-IR spectrum of GO-*g*-AEMA.

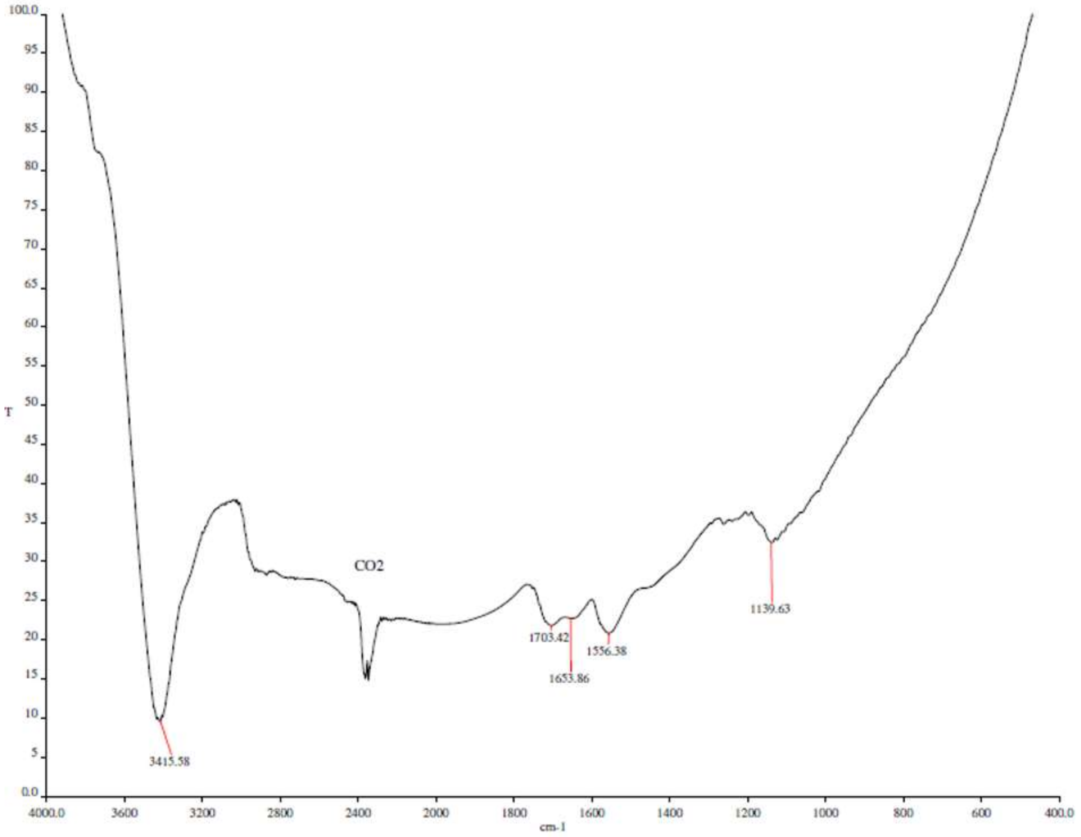

Figure S4. FT-IR spectrum of GO-*g*-poly[DEAEMA]

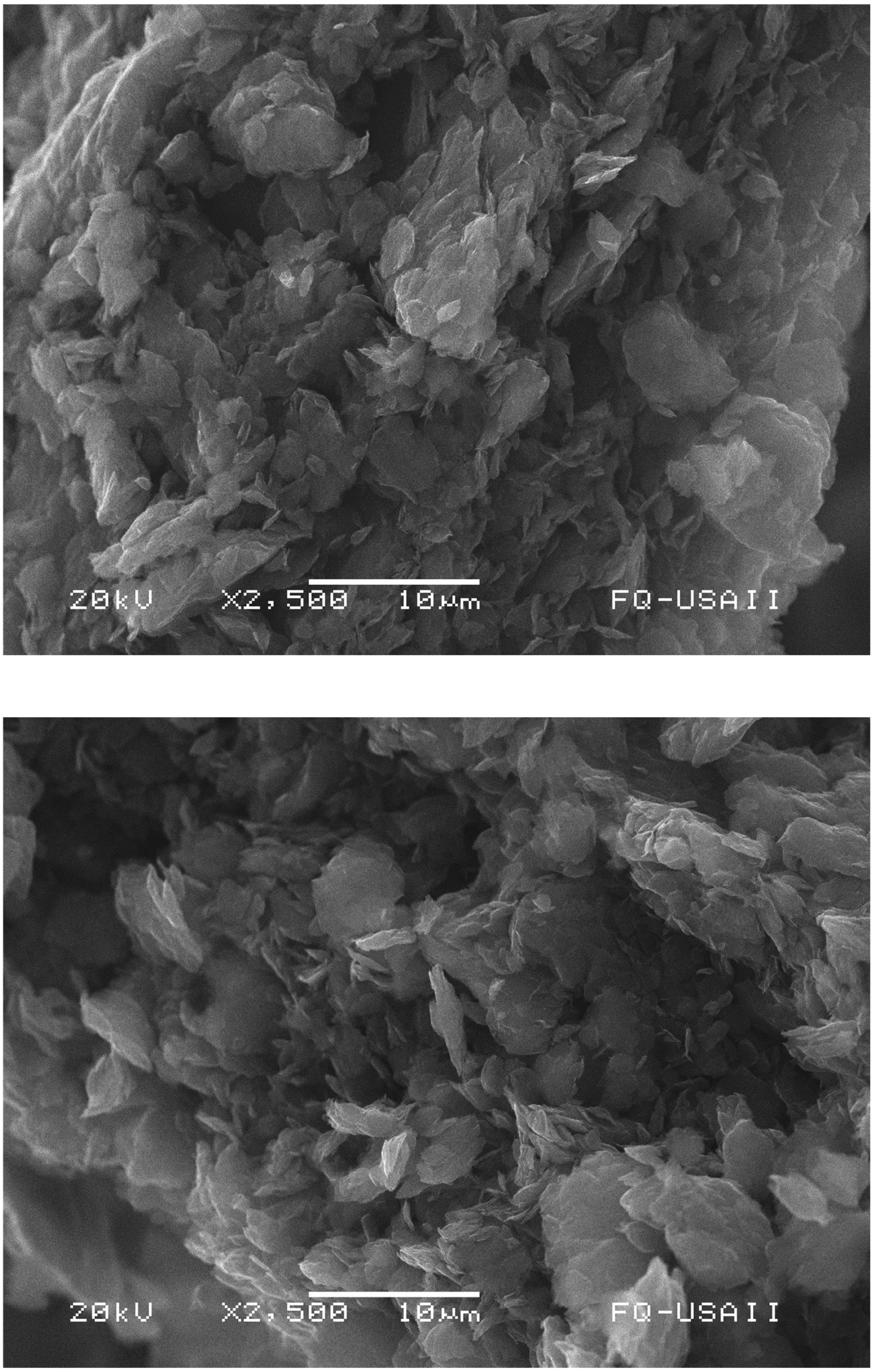

Figure S5. SEM of pristine GO (top) and after synthetic stage I (bottom).

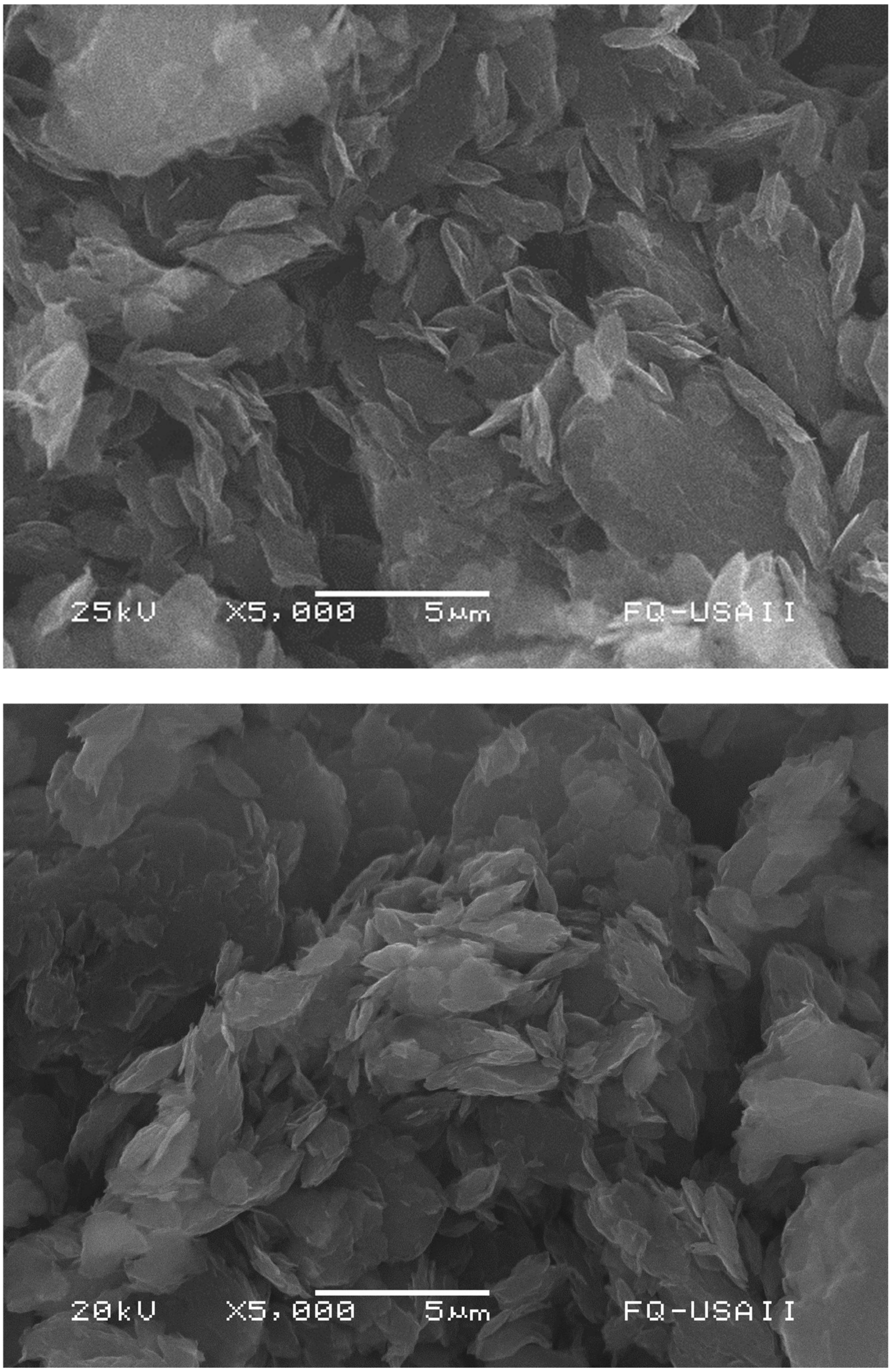

Figure S6. SEM of GO after synthetic stage II (top) and after stage III (bottom).

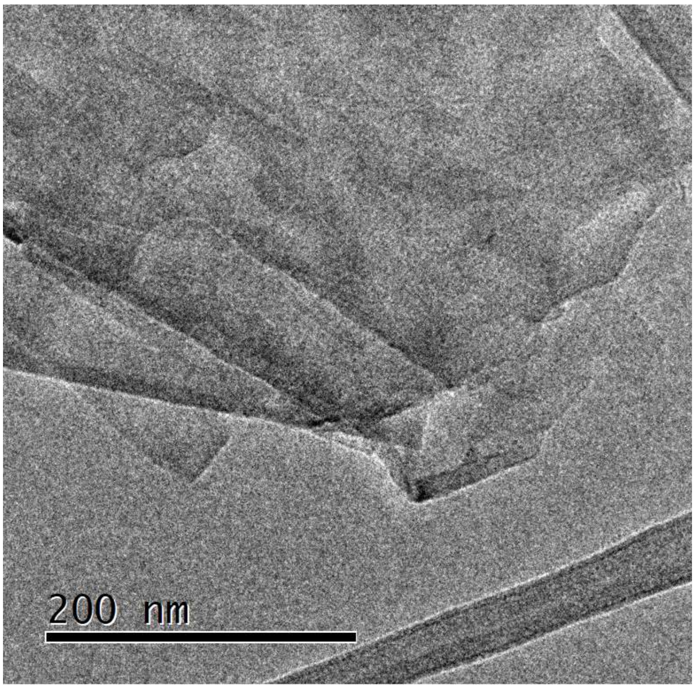

Figure S7. TEM micrograph of GO.

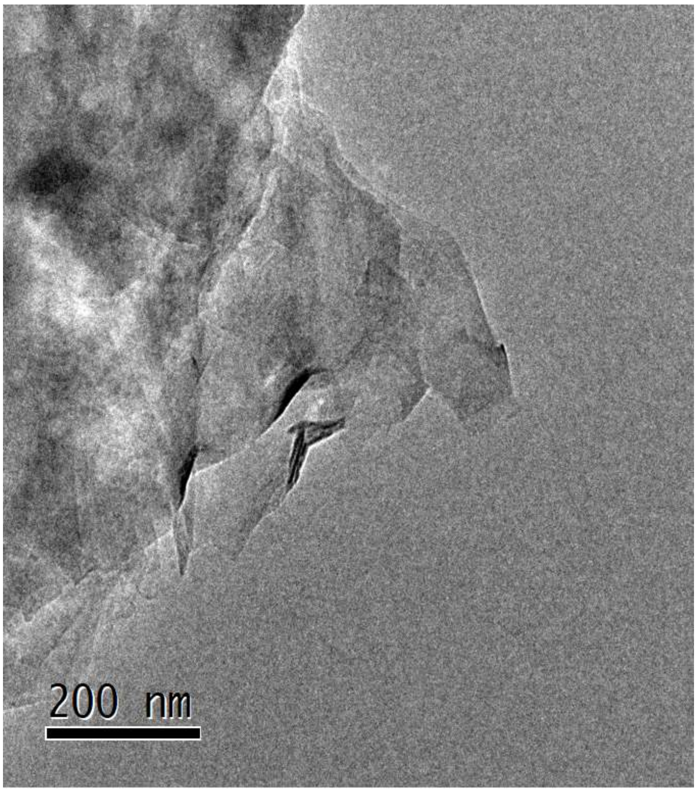

Figure S8. TEM micrograph of GO-*g*-poly[DEAEMA].

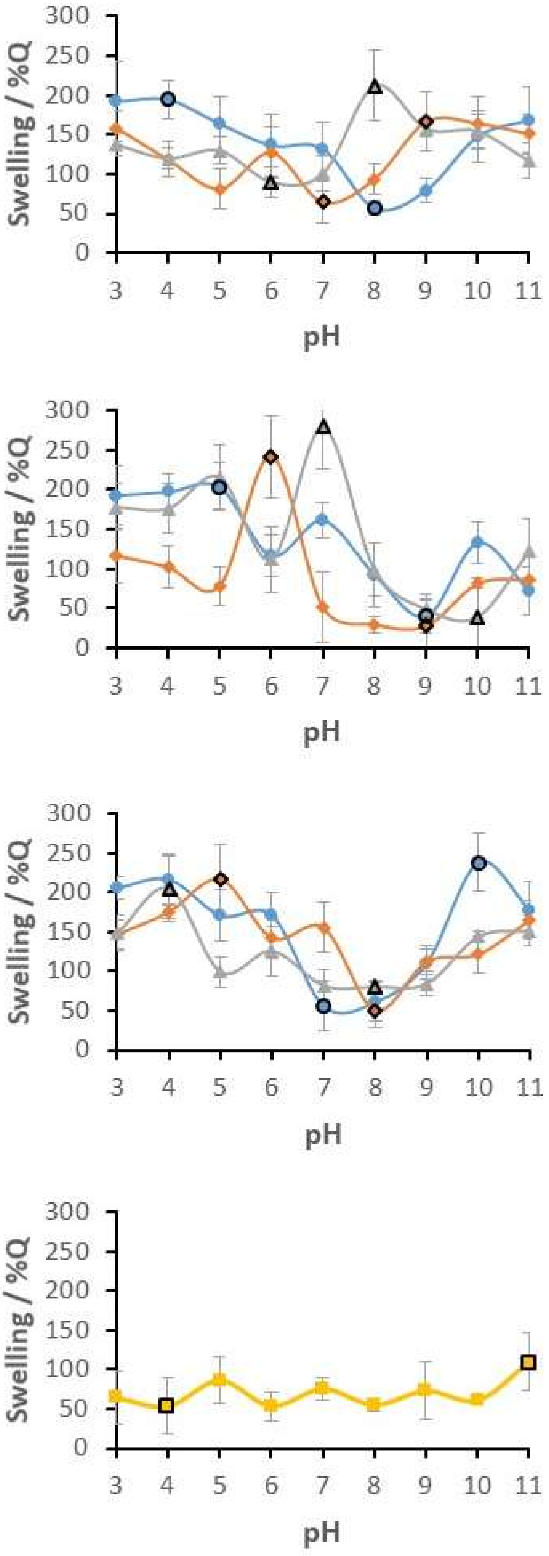

Figure S9. Swelling curves against pH for GO-*g*-poly[DEAEMA] (top three graphs) and blank (graph at the bottom). For GO-*g*-poly[DEAEMA], ratios at stage I (EDC:COOH@GO) are 2 mol/mol (first graph), 4 mol/mol (second graph) and 6 mol/mol (third graph); ratios at stage II (AEMA:GO-*g*-NHS) are 6 mmol/g (blue circles), 12 mmol/g (orange diamonds) and 24 mmol/g (grey triangles). $\%Q_{min}$ and $\%Q_{max}$ are highlighted in each curve. All the curves are set to the same scale in *y* axis for comparative purposes. Average values are for $N = 3$, error bars are 1 s.d.
